# Aerobic training with moderate or high doses of vitamin D improve liver enzymes, LXRα and PGC-1α levels in rats with T2DM

**DOI:** 10.1038/s41598-024-57023-z

**Published:** 2024-03-17

**Authors:** Zahra Hoseini, Nasser Behpour, Rastegar Hoseini

**Affiliations:** Department of Exercise Physiology, Faculty of Sport Sciences, Razi University, P.O.Box. 6714967346, Kermanshah, Iran

**Keywords:** Aerobic training, Vitamin D supplementation, Hepatocytes, Liver enzymes, PGC-1α, LXR α, Type 2 diabetes, Nutrition, Public health, Endocrine system and metabolic diseases, Metabolic disorders

## Abstract

Dysregulation of key transcription factors involved in hepatic energy metabolism, such as peroxisome proliferator-activated receptor gamma coactivator-1 alpha (PGC-1α) and liver X receptor alpha (LXRα), has been observed in T2DM. The present study aims to investigate the effects of aerobic training and vitamin D supplementation on liver enzyme levels and the levels of PGC-1α and LXRα proteins in hepatocytes, in a rat model of T2DM. The study involved 56 male Wistar rats, divided into two groups: one was non-diabetic and acted as a control group (n = 8), and the other had induced diabetes (n = 48). The diabetic rats were then split into six subgroups: two groups received high or moderate doses of vitamin D and aerobic training (D + AT + HD and D + AT + MD); two groups received high or moderate doses of vitamin D alone (D + HD and D + MD); one group underwent aerobic training with vehicle (sesame oil; D + AT + oil), and one group was a diabetic control receiving only sesame oil (oil-receiving). The D + AT + HD and D + HD groups received 10,000 IU of vitamin D, while the D + AT + MD and D + MD groups received 5000 IU of vitamin D once a week by injection. The D + AT + oil group and the sham group received sesame oil. After eight weeks of treatment, body weight, BMI, food intake, serum insulin, glucose, 25-hydroxyvitamin D, ALT, AST, and visceral fat were measured. The levels of PGC-1α and LXRα proteins in the liver was assessed by western blotting. Statistical analysis was performed using the paired t-test, one-way analysis of variance (ANOVA), and the Tukey post hoc test at a significance level of P < 0.05. Body weight, food intake, and BMI decreased significantly in the D + AT + HD, D + AT + MD, D + AT + oil, D + HD, and D + MD groups with the highest reduction being observed in body weight and BMI in the D + AT + HD group. The D + AT + HD group exhibited the lowest levels of insulin, glucose, and HOMA-IR while the D + C group exhibited the highest levels among the diabetic groups. The D + AT + HD and D + AT + MD groups had lower levels of ALT and AST enzymes compared to the other groups with no significant difference between D + AT + HD and D + AT + MD. D + AT + HD (p = 0.001), D + AT + MD (p = 0.001), D + HD (p = 0.023), D + MD (p = 0.029), and D + AT + oil (p = 0.011) upregulated LXRα compared to D + C. Among these groups, D + AT + HD exhibited a more profound upregulation of LXRα than D + AT + MD, D + AT + oil, D + HD, and D + MD (p = 0.005; p = 0.002, p = 0.001, and p = 0.001, respectively). Similarly, D + AT + HD showed a more notable upregulation of PGC-1α compared to D + AT + oil, D + HD, and D + MD (p = 0.002; p = 0.001, and p = 0.001, respectively). Pearson correlation tests showed significant and negative correlations between serum 25-hydroxyvitamin levels and both visceral fat (r = − 0.365; p = 0.005) and HOMA-IR (r = − 0.118; p = 0.009); while positive and significant correlations between the liver-to-bodyweight ratio with both ALT and AST enzymes and also between QUICKI levels with LXRα (r = 0.578; p = 0.001) and PGC-1α (r = 0.628; p = 0.001). Combined administration of aerobic training and vitamin D supplementation potentially improves liver enzymes in type-2 diabetic rats that were simultaneous with upregulating the levels of PGC-1α and LXRα proteins in hepatocytes. These improvements were more significant when combining exercise with high-dose vitamin D supplementation. This study highlights the potential of this combination therapy as a new diabetes treatment strategy.

## Introduction

Type 2 diabetes mellitus (T2DM) is a metabolic disorder characterized by impaired glucose homeostasis and insulin resistance^1^. It is a global epidemic affecting millions of people worldwide and associated with various complications, including cardiovascular disease and Non-Alcoholic Fatty Liver Disease (NAFLD)^2^. As a central organ in energy hemostasis, the liver stores small amounts of fatty acids as triglycerides (less than 5%) under normal circumstances; a mechanism which is altered in the setting of obesity or T2DM leading the hepatic triglycerides accumulation, dyslipidemia, abnormal glucose metabolism^3^.

The proper liver function and the levels of several enzymes are regulated by the activation of lipogenic transcriptional factors such as Liver X Receptors (LXRs) and peroxisome proliferator-activated receptor (PPAR) γ coactivator 1α (PGC1α)^4,5^. The ligand-activated transcription factors, LXRs, belong to the superfamily of nuclear receptor which is activated in the presence of excess cholesterol accumulation with two main subtypes, LXRβ and LXRα that is highly expressed in the liver^6,7^. Once activated, LXRα induces the expression of genes that eliminate the hepatic cholesterol efficiently^8^. In addition, as a transcription factor, PGC-1α regulates lipid oxidation, energy homeostasis, and hepatic fatty acid oxidation^9^.

Currently, apart from lifestyle changes (e.g. physical activity and diet) that can lead to weight loss, no therapies have been more effective for T2DM and NAFLD^10^. Physical activity and Vitamin D (Vit D) supplementation have been demonstrated as effective interventions for managing T2DM^10,11^. Studies suggest that various aerobic training (AT) protocols (i.e. continuous or interval) may lead to fat oxidation and weight loss^12,13^. Studies have shown that AT reduces glycemic indices, liver enzymes, and fatty liver in humans^11^ and rodents^14^. Additionally, serum Vit D levels have been implicated as a potential modulator of glucose homeostasis^14^ and many studies have linked Vit D deficiency to several chronic diseases such as obesity, T2DM, and NAFLD^14–16^.

Previous studies have investigated the effects of both AT^17^ and Vit D supplementation^18^ on glycemic control, insulin action, and cardiorespiratory fitness in individuals with T2DM, reporting contradictory results. Similarly, knowledge about the effects of combined AT and Vit D supplementation with different doses on various metabolic parameters in T2DM is limited. In this study, we investigated the effects of separate and combined AT with different doses of Vit D supplementation on weight, visceral fat, glycemic index, liver enzymes, and protein levels in liver tissue in rats with T2DM. In addition, we explored the associations between the Quantitative Insulin Sensitivity Check Index (QUICKI), levels of PGC-1α and LXRα proteins and also the association between serum Vit D levels with insulin resistance and visceral fat.

## Materials and methods

### Animal preparation

**Figure 1:**
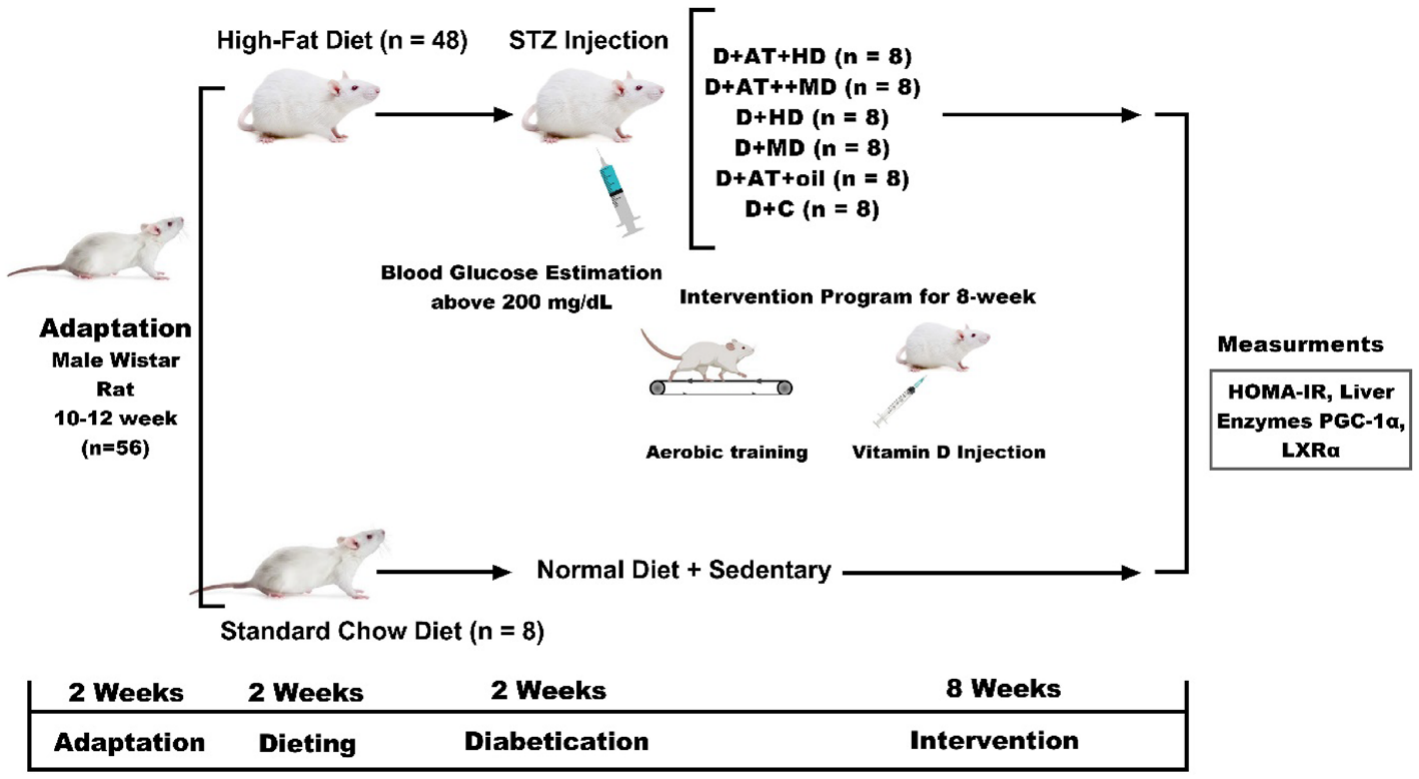
The experimental design of the study. *D + AT + HD* Diabetic + Aerobic Training + High Dose of Vitamin D, *D + AT + MD* Diabetic + Aerobic Training + Moderate Dose of Vitamin D, *HD* Diabetic + High Dose of Vitamin D, *MD* Diabetic + Moderate Dose of Vitamin D, *D + AT + oil* Diabetic + Aerobic Training + Sesame Oil, *D + C* Diabetic + Sesame Oil.

In this laboratory-based experimental study, all procedures were conducted following the Research Ethics Committee of Razi University approved and supervised the study design and conduction (no. IR.RAZI.REC.1401.011), Iran. Animal care, maintenance, and sacrifice have been conducted according to the Danish “Animal Welfare Act” (LBK 1343 of 04/12/2007), and in accordance with ARRIVE guidelines. Fifty-six Wistar rats that were 10–12 weeks old were acquired from the Laboratory Animal Care Center of Medical Sciences University of Kermanshah for use in the study. In order to determine the sample size, based on previous research, a moderate effect size of 0.5, a power level of 0.8, and a significance level of 0.05, with an estimated standard deviation of 1.5, and seven-group design with an equal allocation ratio was used suggesting a total sample size of 48 rats. The animals were housed in polycarbonate cages that were transparent and exposed to ambient temperatures of 21 ± 2 °C, relative humidity of 45–55%, and a dark–light cycle of 12:12 h. They were provided with standard mouse food for sustenance and given water in 500 mL bottles. Throughout the study, the animals were granted unrestricted access to both food and water, in compliance with established laboratory animal procedures at the University of Kermanshah. After a two-week adaptation period to the environment, the rat standard chow diet was exchanged for a High-Fat Diet (HFD). The HFD consisted of a mixture of standard mouse food powder (365 mg/kg), mixed vitamins and minerals (60 mg/kg), yeast powder (1 mg/kg), sheep fat (310 mg/kg), DL-methionine (3 mg/kg), and sodium chloride (1 mg/kg) in pellet form, which was purchased from Beh-Parvar Company. This specific diet has been shown in previous studies to induce obesity in rats^19,20^. After a weight gain of over 300 g, the rats were divided into two main groups: sham (non-diabetic control, n = 8) and diabetic (n = 48). The rats were then given a nicotinamide solution at a dosage of 110 mg/kg body weight, followed by an injection of streptozotocin solution dissolved in citrate buffer at a dosage of 50 mg/kg body weight (pH 4.5) 15 min later. Using this procedure, diabetes was induced in the rats, which was later confirmed by obtaining a small drop of blood from a tail incision made by a Lancet. The blood was transferred to a glucometer strip and blood glucose levels exceeding 200 mg/dL served as a metric to confirm type 2 diabetes in the rats. No insulin treatments were administered to the animals throughout the study (Fig. 1).

### Groups and vitamin D supplementation

Following diabetes induction, the rats were divided into seven groups, including one sham group (sham; n = 8) and six diabetic groups. The latter groups were further divided into D + AT + HD (diabetic with high dose and exercise), D + AT + MD (diabetic with moderate dose and exercise), D + HD (diabetic with high dose), D + MD (diabetic with moderate dose), D + AT + oil (diabetic with exercise and vehicle), and D + C (diabetic control). Each of the diabetic groups received weekly co-treatment with either high (HD; 10,000 IU/kg), moderate (MD; 1000 IU/kg), or sesame oil. Based on the composition of the animal's diet (5000 IU/kg food), each rat in the HD and MD groups received approximately 100 IU of Vit D from food, with the remainder supplied via weekly injections^14^.

### Aerobic training

The intensity of AT performed by the rats during the study was proportional to their speed on the treadmill and was monitored throughout. A running speed range of 20–25 m/min was determined to be the appropriate level of AT that would elicit a level of intensity equivalent to 70 to 75% of maximum oxygen consumption in rats^21^. The study employed a training program consisting of AE on a treadmill for 5 days a week for 8 weeks. During the first two weeks of the training program, the AT started at a speed of 10 m/min in 15 min intervals, which were then gradually increased in both intensity and duration per week. The last two weeks involved the highest intensity level of 25 m/min, with the duration of the activity reaching 30 min, which remained consistent with the same 0°-degree slope of the treadmill throughout the training period. A warm-up period of 5 min, with a speed of 5 to 10 m/min, was performed before each training session, and a similar cooling period was employed after each session^22^. This study employed the ASCM principles concerning scientific training protocol design (Table 1).

**Table 1:** Numerical representation of the protocol in different weeks.

Week	Acquaintance	1st	2nd	3rd	4th	5th	6th	7th	8th
Exercise duration (min)	5	15	15	20	20	25	25	30	30
Rolling speed (m/min)	10	10	10	15	15	20	20	25	25

### Body weight, body mass index, waist circumference, and food intake

The animals were weighed once a week, specifically between 08:00 h and 10 h, using a Sartorius (Germany) scale. Body length was measured from the nose to the anus, and body mass index (BMI) was calculated. To evaluate Food Intake (FI), identical amounts of food were given to each animal in its respective cage (30 g/day). The amount consumed was quantified by subtracting the weight of the remaining food from the total amount provided.

### Blood sampling, visceral fat, and histochemical analyses

After the final training session, rats were anesthetized via an intraperitoneal injection of xylazine and ketamine (40 mg/kg body weight) 48 h later. Full anesthesia was confirmed by the loss of pedal and corneal reflexes, and blood samples were collected by exposing the vena cava. The collected blood was separated from the sample through decapitation, followed by centrifugation at 4000×*g* for 10 min, with the supernatant being used to measure levels of glucose, insulin, and serum 25(OH)D concentration. Concentrations of serum glucose and insulin were measured using enzymatic (GOD-PAP glucose oxidase aminoantipyrine) colorimetric method (Pars Azmoun, Tehran, Iran) and a rat insulin ELISA kit (DRG, Springfield Township, NJ, USA), respectively. The coefficients of variation were 1.62% and the sensitivity of the method for insulin was 1.76 mg/dL. Homeostatic model assessment, as an index of insulin resistance (HOMA-IR) and QUICKI were calculated from the following equations:

$$ ({\text{fasting}}\;{\text{insulin }}\left[ {{\text{mU}}/{\text{mL}}} \right) \times {\text{fasting}}\;{\text{ glucose }}\left[ {{\text{mmol}}/{\text{L}}} \right]/{22}.{5}) $$

$$ {\text{QUICKI}} = {1}/[{\text{log}}\left( {{\text{I}}0} \right) + {\text{log}}\left( {{\text{G}}0} \right)]. $$

The Alanine Aminotransferase (ALT) and Aspartate Aminotransferase (AST) levels were determined using enzymatic kits from Pars Azmoon (1-400-019 and 1-400-018), with measurements made according to the methods recommended by the International Federation of Clinical Chemistry and Laboratory Medicine (IFCC). This particular method possesses the capacity to measure changes in photon absorption up to 0.16 at a wavelength of 340 nm. Also, visceral fat was measured directly by weightening the retroperitoneal fat mass. After performing dissection and extraction of the retroperitoneal fat mass from the rats, the retroperitoneal fat depot located behind the peritoneum and surrounding the kidneys was removed carefully. Then, the isolated retroperitoneal fat mass was assessed using a precision balance for weight measurement (Sartorius scale, Germany).

### Liver isolation and immunoblotting

To isolate the liver using the enzyme digestion method, rats were anesthetized and the liver was exposed surgically by making a small incision at the midline of the abdomen. The excised liver was immediately placed in an ice-cold digestion buffer dish, minced, and transferred to a conical tube containing digestion buffer (containing 100 sodium fluoride, 100 sodium pyrophosphate, 10 sodium vanadate, 10 EDTA, 0.1 mg of aprotinin/ml, and 2 PMSF; pH 7.4, mM; 1% Triton-X 100, 100 Tris). To encourage enzyme digestion, the tube was gently shaken at regular intervals in a shaking water bath at 37 °C for 20–30 min. Then, the digested liver tissue was filtered through a 70-μm cell strainer and centrifuged at 50×*g* for 1 min. The supernatant was subsequently centrifuged at 50×*g* for 1 min and suspended in PBS buffer with protease inhibitors for downstream analyses. For western blotting, the liver homogenates were incubated on ice for 30 min mixed with RIPA buffer and then centrifuged at 12,000 rpm for 10 min at 4 °C. The supernatant was transferred to a tube and incubated overnight with anti-PGC-1α or anti-LXRα antibody at 4 °C. Then, 50 μL of Protein G Sepharose beads were added to the samples, incubated for 2 h at 4 °C, centrifuged at 3000 rpm for 5 min, and washed with RIPA buffer. The samples were then boiled in an SDS-PAGE sample buffer for 10 min. During the gel electrophoresis stage, the proteins were separated on an SDS-PAGE gel. The gel was prepared by pouring a resolving gel and a stacking gel with appropriate concentrations of acrylamide and bis-acrylamide, as well as a suitable running buffer. The protein samples were loaded into wells in the stacking gel and an electric field was applied, causing the proteins to migrate through the gel based on their size. The gel electrophoresis was run at a constant voltage or current for a specific duration to achieve optimal separation of the proteins. After the gel electrophoresis, the proteins were transferred from the gel to a PVDF membrane through a process called electroblotting. The gel was sandwiched between filter paper and the membrane, and an electric current was applied. This facilitated the transfer of the proteins from the gel onto the membrane, preserving their relative positions. Following the transfer, the membrane was blocked with a blocking buffer to prevent nonspecific binding of antibodies. After blocking, the membrane was incubated with primary antibodies (anti-PGC-1α or anti-LXRα antibodies) that specifically recognize the target proteins. The primary antibodies bound to their respective target proteins on the membrane. For detection of the primary antibodies, secondary antibodies were used. These secondary antibodies are labeled with enzymes or fluorophores that allow for visualization of the protein bands. The secondary antibodies bind to the primary antibodies, amplifying the signal and enabling the detection of the target proteins. To visualize the protein bands, chemiluminescence detection reagents were applied to the membrane. The chemiluminescent reaction produced light, which was captured using a chemiluminescence imaging system. The system recorded the emitted light as an image, allowing for the visualization and analysis of the protein bands on the membrane.

### Statistical analysis

The normality of the data was assessed using the Shapiro–Wilk test. Paired data were subsequently analyzed with paired t-tests for pre and post analyse of body weight, body mass index, waist circumference, and food intake and one-way ANOVA with the Tukey test for group comparisons. Results were considered significant at P < 0.05 for all statistical evaluations. Data were analyzed using SPSS version 26 and presented as mean ± SD.

### Ethical approval

All actions performed on the animals were following the guidelines of the Ethics Committee of the Razi University of Kermanshah (IR.RAZI.REC.1401.011 on 27/06/2022) and in accordance with ARRIVE guidelines.

## Results

Table 2 summarizes the changes in body weight, FI, and BMI between diabetic rats (n = 48) and SHAM (n = 8). Significant differences in body weight, FI, and BMI were observed between diabetic rats and SHAM at the onset and after eight weeks of intervention. Body weight, FI, and BMI decreased significantly in the D + AT + HD, D + AT + MD, D + AT + oil, D + HD, and D + MD groups at the end of the study compared to the beginning, with the highest reduction occurring in the D + AT + HD group (p < 0.05 for all). Notably, a significant difference was observed in the average body weight and BMI among all groups, but no significant differences were found between FI of the D + AT + HD with D + AT + MD (p = 0.434); and between D + AT + MD with D + HD (p = 0.133) and D + AT + oil (p = 0.905) groups; and between D + HD with D + MD (p = 0.836) and D + AT + oil groups (p = 0.748), and between D + MD with D + AT + oil group (p = 0.902).

**Table 2:** Comparison of mean ± SD of body weight, FI, and BMI before and after intervention.

Variables	D + AT + HD	D + AT + MD	D + HD	D + MD	D + AT + oil	D + C	SHAM	P-value^a^
Body weight (g)								
Before	316.87 ± 1.72	316.87 ± 1.72	308.75 ± 2.60	306 ± 2.50	313.87 ± 1.88	313.87 ± 1.88	224.12 ± 4.96	
After	281.62 ± 2.06	286.62 ± 2.38	293.37 ± 2.55	295.62 ± 2.26	293.50 ± 2.20	293.50 ± 2.20	228.87 ± 5.27	
P^†^	0.001*	0.001*	0.001*	0.001*	0.001*	0.001*	0.001*	
Δ	− 35.25 ± 0.462^A^	− 30.25 ± 2.434^B^	− 15.37 ± 0.517^D^	− 10.37 ± 0.744^E^	− 20.37 ± 0.517^C^	16.75 ± 0.464^G^	4.75 ± 0.460^F^	0.001^¥^
FI (g/day)								
Before	20.25 ± 2.12	18.87 ± 1.35	17.25 ± 1.48	16.75 ± 1.48	18.12 ± 2.16	19.87 ± 1.88	12.75 ± 1.66	
After	16.37 ± 1.68	15.75 ± 1.28	15.12 ± 1.24	15.12 ± 1.52	15.43 ± 2.63	22.62 ± 1.76	13.37 ± 1.50	
P^†^	0.001*	0.001*	0.003*	0.013*	0.002*	0.001*	0.049*	
Δ	− 3.87 ± 1.246^A^	− 3.12 ± 0.640^AB^	− 2.12 ± 0.640^BCDE^	− 1.62 ± 0.231^CDE^	− 2.68 ± 0.883^BC^	2.75 ± 0.462 ^G^	0.62 ± 0.744^F^	0.001^¥^
BMI (kg/m^2^)								
Before	0.78 ± 0.138	0.79 ± 0.061	0.83 ± 0.068	0.75 ± 0.083	0.81 ± 0.059	0.81 ± 0.105	0.60 ± 0.062	
After	0.70 ± 0.122	0.72 ± 0.056	0.79 ± 0.064	0.72 ± 0.081	0.76 ± 0.056	0.85 ± 0.111	0.61 ± 0.064	
P^†^	0.001*	0.002*	0.004*	0.011*	0.003*	0.004*	0.046*	
Δ	− 0.08 ± 0.0158^A^	− 0.06 ± 0.005^B^	− 0.04 ± 0.003^D^	− 0.02 ± 0.002^E^	− 0.05 ± 0.004^C^	0.04 ± 0.006^G^	0.01 ± 0.001^F^	0.001^¥^

*BMI* Body Mass Index, *FI* Food Intake, *WC* Waist Circumference, *D + AT + HD* Diabetic + Aerobic Training + High Dose of Vitamin D, *D + AT + MD* Diabetic + Aerobic Training + Moderate Dose of Vitamin D, *HD* Diabetic + High Dose of Vitamin D, *MD* Diabetic + Moderate Dose of Vitamin D, *D + AT + oil* Diabetic + Aerobic Training + Sesame Oil, *D + C* Diabetic + Sesame Oil, *SHAM* Non-Diabetic Control.

Data analysis was done by the analysis of one-way analysis of variance test followed by post hoc Tukey's test; *P*^*†*^ Statistical analysis was done by paired sample t-test; *Significantly different in comparison pre and post-within the groups; P-value^a^Statistical analysis was done by one-way analysis test; ^¥^Significantly different comparing Δ between groups.

The mean values followed by different letters (A, B, C, D, E, F, and G) mean significantly different at the 0.05 level (p < 0.05). The values followed by the same letter are not significantly different. Dissimilar letters represent a significant difference between the groups.

**Figure 2:**
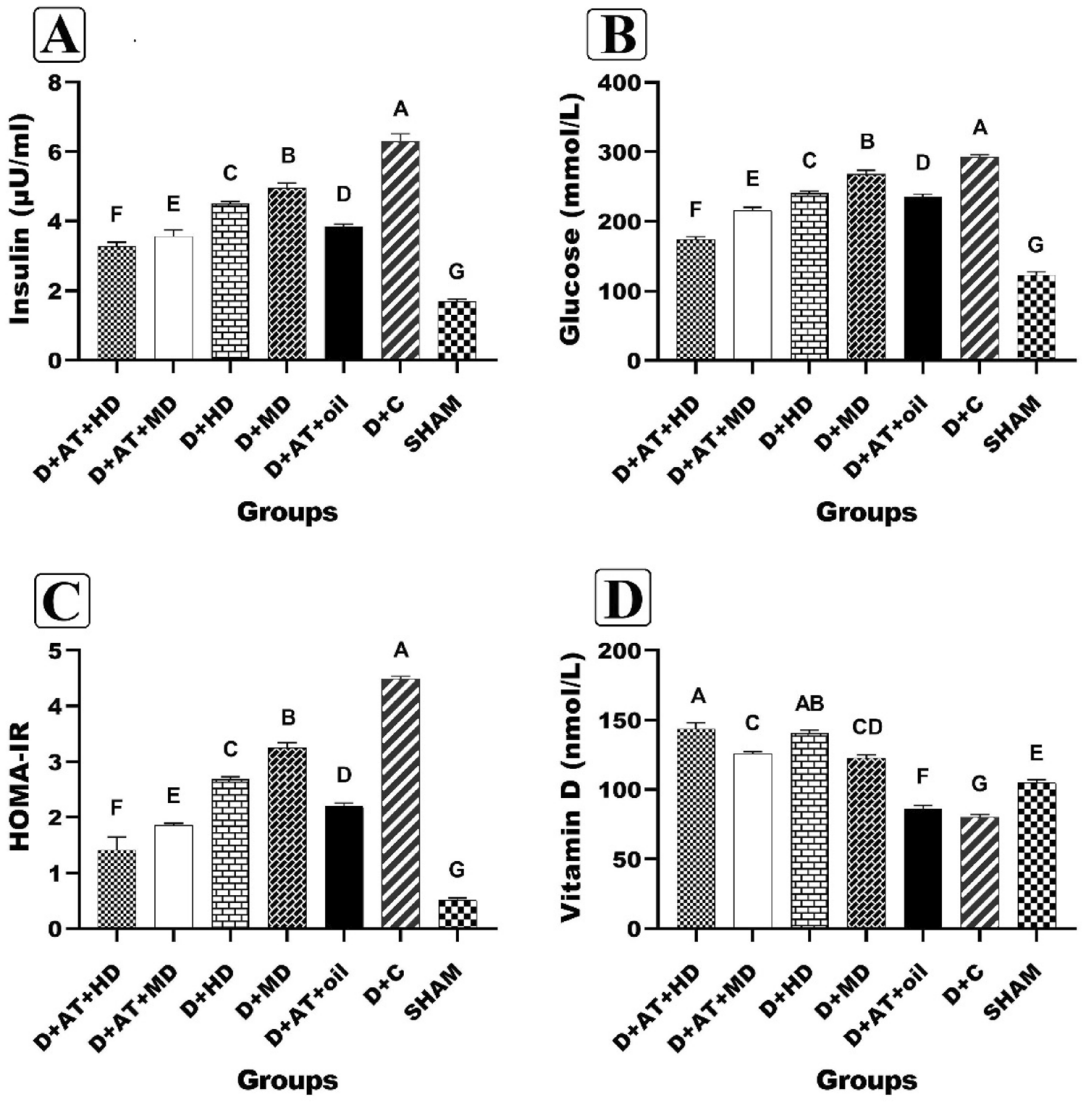
Comparison between mean ± SD of insulin (**A**), glucose (**B**), HOMA-IR (**C**), and serum 25-hydroxyvitamin D (**D**) between groups. *D + AT + HD* Diabetic + Aerobic Training + High Dose of Vitamin D, *D + AT + MD* Diabetic + Aerobic Training + Moderate Dose of Vitamin D, *HD* Diabetic + High Dose of Vitamin D, *MD* Diabetic + Moderate Dose of Vitamin D, *D + AT + oil* Diabetic + Aerobic Training + Sesame Oil, *D + C* Diabetic + Sesame Oil, *SHAM* Non-Diabetic Control. The mean values followed by different letters (A, B, C, D, E, F, and G) mean significantly different at the 0.05 level (p < 0.05). The values followed by the same letter are not significantly different. Dissimilar letters represent a significant difference between the groups.

Figure 2 presents significant variations in insulin, glucose, HOMA-IR, and serum 25-hydroxyvitamin D between the diabetic and SHAM groups (A, B, C, and D). Additionally, there were significant differences in insulin, glucose, and HOMA-IR between diabetic groups, with the D + AT + HD group exhibiting the lowest levels and the D + C group the highest. The study results indicate a statistically significant difference in serum 25-hydroxyvitamin D levels between diabetic groups, except for the D + HD and D + MD groups (P = 0.189), with the highest levels in the D + AT + HD group and the lowest in the D + C group. Furthermore, D + AT + HD, D + AT + MD, D + HD, D + MD, and D + AT + oil groups showed significantly higher serum 25-hydroxyvitamin D levels compared to the D + C group (P < 0.05 for all three variables).

**Figure 3:**
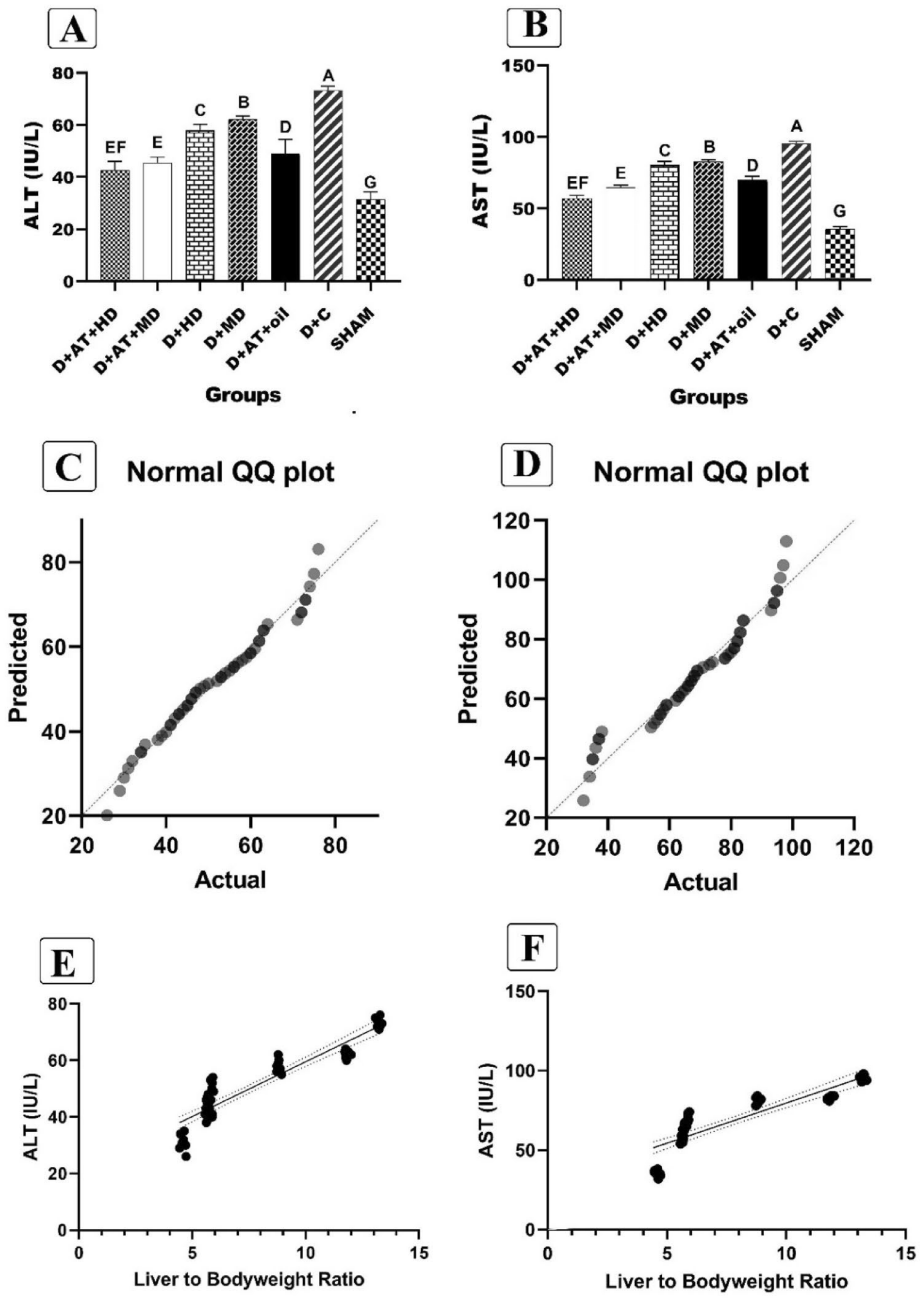
Comparison between mean ± SD of ALT and AST between groups (**A**, **B**); Checking the normal distribution liver-to-bodyweight ratio and ALT and AST enzymes (**B**, **C**); The relationship between the liver-to-bodyweight ratio and ALT and AST enzymes (**C**, **D**). *D + AT + HD* Diabetic + Aerobic Training + High Dose of Vitamin D, *D + AT + MD* Diabetic + Aerobic Training + Moderate Dose of Vitamin D, *HD* Diabetic + High Dose of Vitamin D, *MD* Diabetic + Moderate Dose of Vitamin D, *D + AT + oil* Diabetic + Aerobic Training + Sesame Oil, *D + C* Diabetic + Sesame Oil, *SHAM* Non-Diabetic Control. Data analysis was done by the analysis of one-way analysis of variance test followed by post hoc Tukey's test; and Pearson correlation test. The mean values followed by different letters (A, B, C, D, E, F, and G) mean significantly different at the 0.05 level (p < 0.05). The values followed by the same letter are not significantly different. Dissimilar letters represent a significant difference between the groups.

The data presented in Fig. 3A and B showed that the D + AT + HD and D + AT + MD groups had lower levels of ALT and AST enzymes compared to other groups. No significant difference was observed between the D + AT + HD and D + AT + MD groups (p = 0.375) regarding the levels of these enzymes. However, a significant difference was observed in ALT and AST enzymes between other groups (P < 0.05 for all variables). Moreover, Fig. 3B and C presents the normal distribution of liver-to-bodyweight ratio and ALT (p = 0.185) and AST (p = 0.093) enzymes. The Pearson correlation test conducted in Fig. 3E and F demonstrated a positive and significant correlation between the liver-to-bodyweight ratio and both ALT (r = 0.925; p = 0.001) and AST (r = 0.874; p = 0.009).

**Figure 4:**
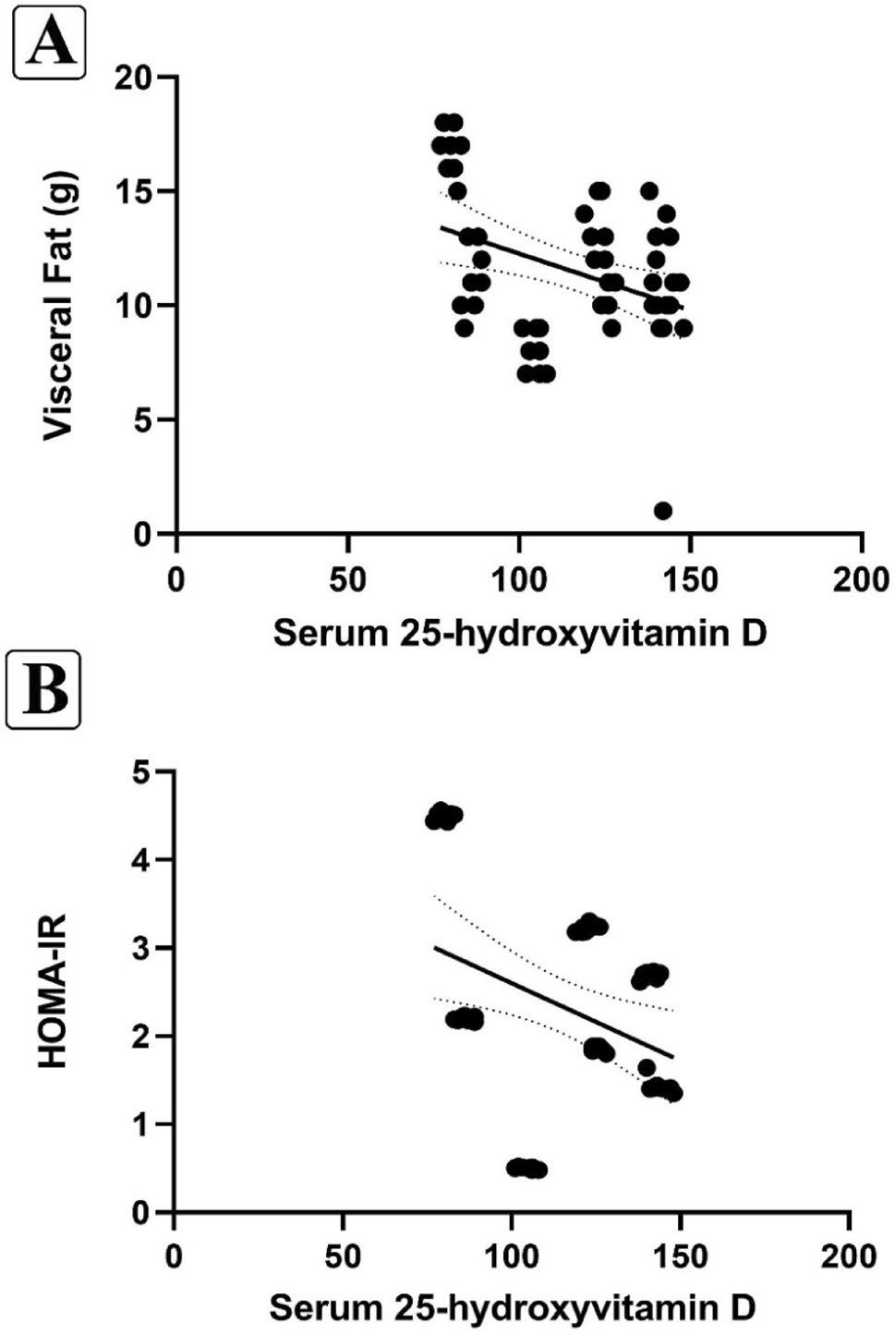
The relationship between the serum 25-hydroxyvitamin with visceral fat (**A**), and HOMA-IR (**B**). Data analysis was done by Pearson correlation test at the 0.05 level (p < 0.05).

As presented in Fig. 4A and B, Pearson's correlation coefficient test revealed a significant and negative correlation between serum 25-hydroxyvitamin levels and both visceral fat (r = − 0.365; p = 0.005) and HOMA-IR (r = − 0.118; p = 0.009).

**Figure 5:**
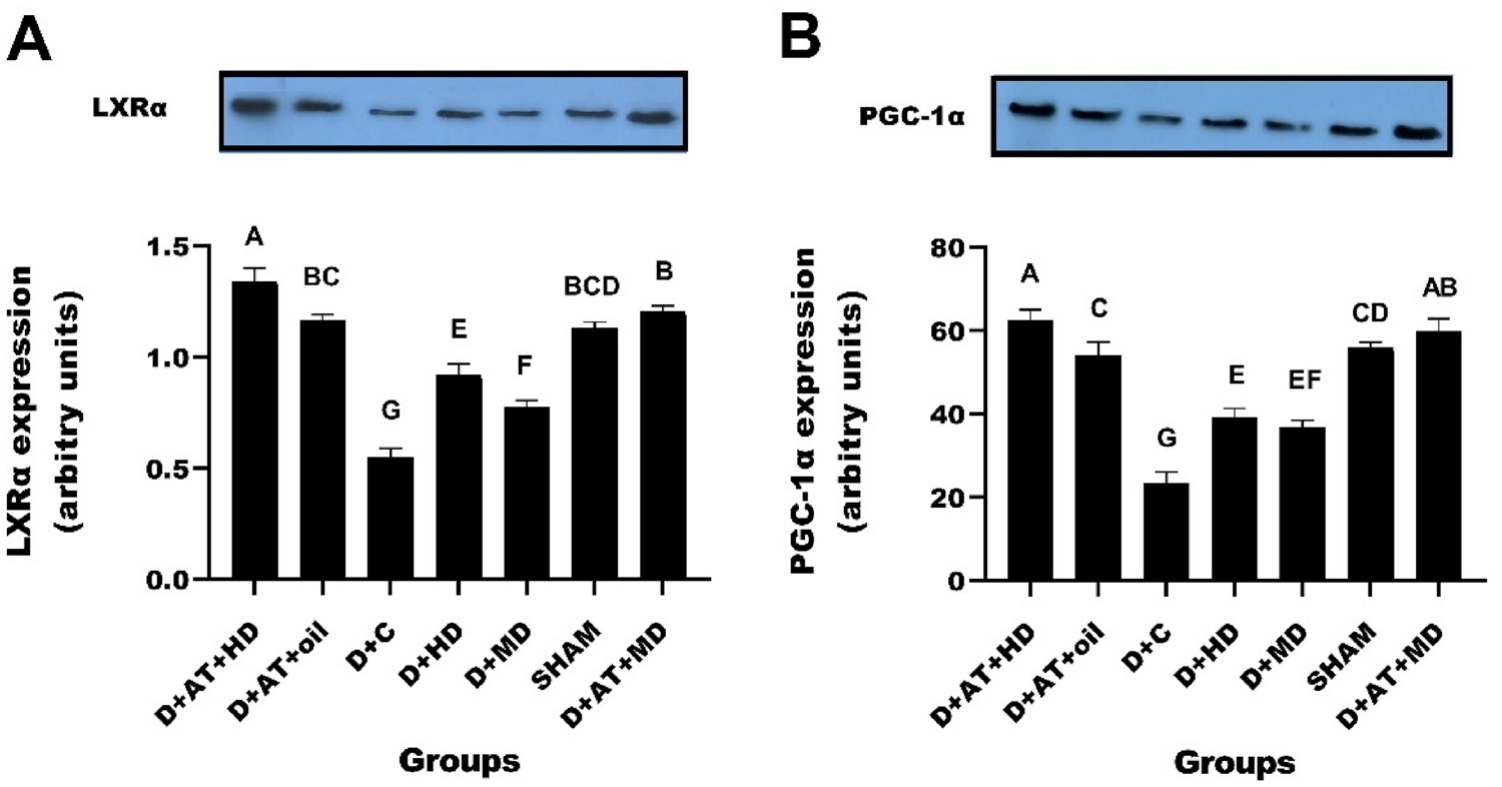
Comparison between mean ± SD of LXRα (**A**) and PGC-α (**B**) between groups. *D + AT + HD* Diabetic + Aerobic Training + High Dose of Vitamin D, *D + AT + MD* Diabetic + Aerobic Training + Moderate Dose of Vitamin D, *HD* Diabetic + High Dose of Vitamin D, *MD* Diabetic + Moderate Dose of Vitamin D, *D + AT + oil* Diabetic + Aerobic Training + Sesame Oil, *D + C* Diabetic + Sesame Oil, *SHAM* Non-Diabetic Control. Data analysis was done by the analysis of one-way analysis of variance test followed by post hoc Tukey's test. The mean values followed by different letters (A, B, C, D, E, F, and G) mean significantly different at the 0.05 level (p < 0.05). The values followed by the same letter are not significantly different. Dissimilar letters represent a significant difference between the groups.

Figure 5A and B portray the significant differences in levels of PGC-1α and LXRα proteins between the diabetic and SHAM groups. One-way ANOVA revealed significant differences in the level of these proteins between diabetic groups. Moreover, D + AT + HD (p = 0.001), D + AT + MD (p = 0.001), D + HD (p = 0.023), D + MD (p = 0.029), and D + AT + oil (p = 0.011) upregulated LXRα compared to D + C. Among these groups, D + AT + HD exhibited a more profound upregulation of LXRα than D + AT + MD, D + AT + oil, D + HD, and D + MD (p = 0.005; p = 0.002, p = 0.001, and p = 0.001, respectively). Conversely, no significant differences in LXRα levels were observed between D + AT + MD, D + AT + oil, and SHAM (Fig. 5A). Similarly, D + AT + HD showed a more notable upregulation of PGC-1α compared to D + AT + oil, D + HD, and D + MD (p = 0.002; p = 0.001, and p = 0.001, respectively). No significant differences in PGC-1α levels were observed between D + AT + HD and D + AT + MD (p = 0.326), D + AT + oil and SHAM (p = 0.729), and D + HD and D + MD (p = 0.452) (Fig. 5B).

**Figure 6:**
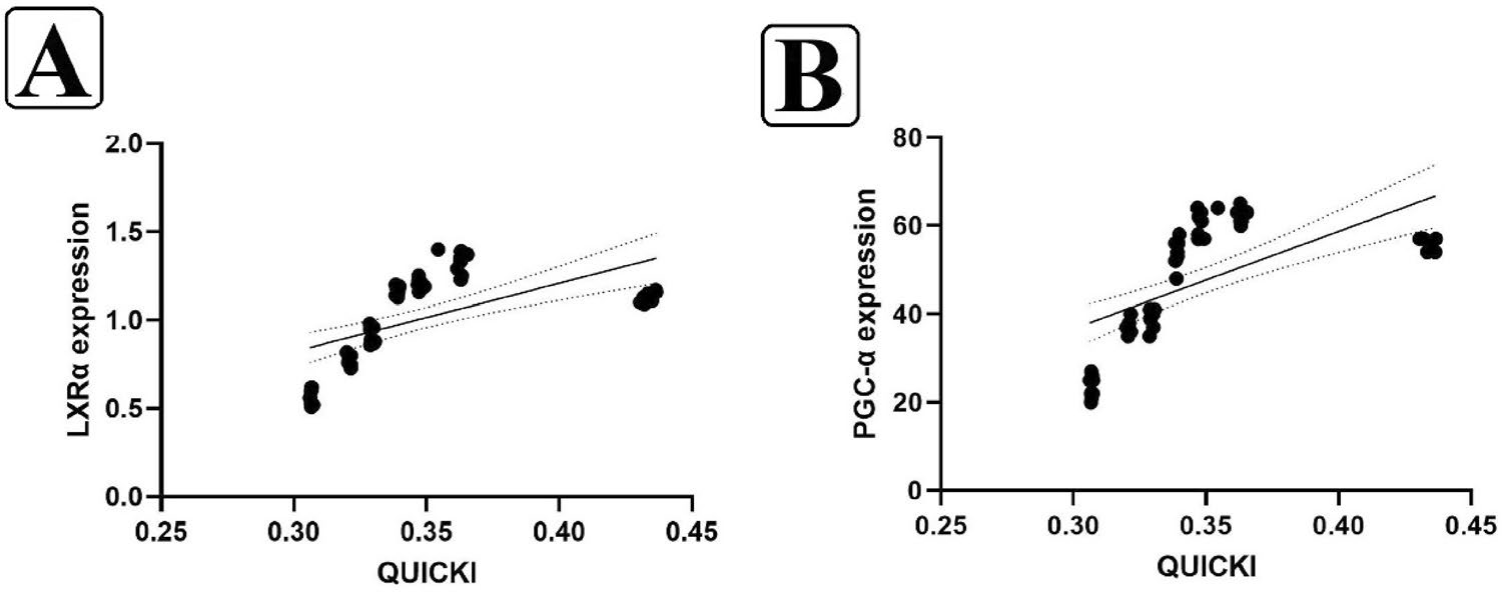
The relationship between the QUICKI with LXRα (**A**) and PGC-α (**B**). Data analysis was done by Pearson correlation test at the 0.05 level (p < 0.05).

As demonstrated in Fig. 6A and B, Pearson's correlation coefficient analysis revealed a significant and positive correlation between QUICKI levels and both LXRα (r = 0.578; p = 0.001) and PGC-1α (r = 0.628; p = 0.001).

## Discussion

Our findings in HFD + STZ rats provide direct evidence for the antidiabetic role of AT and Vit D supplementation as suspected before^19,23^. Our results suggest that weight, visceral fat, and the ratio of liver to body weight decreased in D + AT + HD, D + AT + MD, D + AT + oil, D + HD, and D + MD compared to the D + C group, confirming previous findings^20,24^. In line with our results, similar decreases in body weight, visceral fat, BMI, and FI were confirmed in rats with metabolic syndrome following eight-week AT with Vit D^23^ and in human subjects following exercise^25^. AT has been shown to increase the expression of genes involved in energy metabolism and fat oxidation, while also reducing the expression of genes involved in adipogenesis^26,27^. Vitamin D has been found to regulate calcium homeostasis, which is crucial for proper energy metabolism and adipogenesis^21^. It is also believed that both interventions may affect appetite-regulating hormones^28,29^. Furthermore, Vit D supplementation has been linked to increased levels of serotonin, a neurotransmitter that can reduce appetite^28^.

Histochemical blood analyses reveal that in D + AT + HD, D + AT + MD, and D + AT + oil groups there are improved glycemic indices. Previous studies have suggested that AT facilitates the activation of AMP-Activated Protein Kinase (AMPK) that induces the diffusion of Glucose Transporter Type 4 (GLUT4) in muscle by activating signaling pathways that stimulate TBC1D1, thus increasing more efficient glucose uptake and insulin sensitivity^30^. Long-term AT ameliorates the pathophysiologic pathways involved in insulin resistance reducing adipokines, and inflammatory and oxidative stress responses leading to improved insulin sensitivity^31^. Also, AT can lead to changes in protein expression related to glucose metabolism and mitochondrial biogenesis, further improving glycemic control. Our results also suggest a therapeutic effect for high doses of Vit D supplementation in the diabetic state as previously reported by other studies^14,32^. Specifically, it has been suggested that Vit D may stimulate insulin secretion by reducing inflammation, oxidative stress, and apoptosis in pancreatic beta cells, and increasing the intracellular calcium levels. Additionally, Vit D improves insulin resistance by increasing the binding of the active form of Vit D, calcitriol, to Vitamin D Receptor (VDR), forming a heterodimer with the Retinoid-X Receptor (RXR) leading to the modulation of several gene expressions involved in glucose metabolisms, such as PPARγ, Hepatocyte Nuclear Factor-1 Alpha (HNF-1α), and insulin receptor substrates (IRs) as a transcription factor^14^.

We recently showed a marked reduction in the liver enzymes following eight-week AT and Vit D supplementation in human subjects of NAFLD^33^. Accordingly, we found a significantly lower liver enzyme level in D + AT + HD, D + AT + MD, D + AT + oil, D + HD, and D + MD compared to D + C in the present study. Consistent with our results, a reduction of AST and ALT had been reported previously in human subjects of T2DM^34^. Similar to our results, some studies have linked the exercise-induced alleviation of liver enzymes with a significant reduction in hepatic lipid accumulation and whole-body fat mass. However, there were reports of improved liver enzymes without any considerable weight loss after AT^35^. AT improves liver function in rats with T2DM through several cellular and molecular mechanisms including the activation of AMPK, increasing fatty acid oxidation in the liver, increasing antioxidant enzyme activity, and reducing pro-inflammatory cytokine expression leading to reduced oxidative stress, inflammation, and hepatic steatosis^36^. Additionally, Vit D supplementation improved liver enzymes probably by regulating the expression of enzymes involved in glucose metabolism reducing inflammation in the liver, improving mitochondrial function, which can help prevent oxidative stress and damage to liver cells, and inhibiting the production of cytokines^37,38^.

As shown by immunoblotting D + AT + HD, D + AT + MD, and D + AT + oil groups showed relatively upregulated PGC-1α and LXRα in hepatocytes of diabetic rats. Significantly higher level of LXRα has been reported in recent studies following AT program compared to sedentary diabetic rats while there are still some contradictory results. Increased demand for energy during AT boosts oxidative phosphorylation that might trigger a cascade of molecular signaling pathways that ultimately lead to the upregulation of PGC-1α levels^9,39^. The exact mechanisms of increased LXRα levels following AT are complex and multifactorial including the increment of high-density lipoprotein (HDL) cholesterol, AMPK, and PGC-1α activity, which in turn promotes lipid metabolism in the liver and activates LXRα^8,39^. Furthermore, recent studies have suggested that AT may also activate LXRs through direct interaction with certain fatty acids and other molecules involved in lipid metabolism^40^.

Moreover, Vit D supplementation appears to affect PGC-1α and LXRα levels through a combination of direct and indirect mechanisms involving VDRs, co-activators, calcium homeostasis, and lipid metabolism leading to activated downstream signaling pathways that ultimately leads to increased PGC-1α and LXRα levels^41,42^. More research is needed to fully understand the mechanisms by which Vit D supplementation affects PGC-1α and LXRα levels.

The study demonstrated that the groups receiving aerobic training and high doses of vitamin D (D + AT + HD) exhibited lower levels of liver enzymes ALT and AST compared to other groups. This suggests that the intervention may have a positive effect on liver health in rats with T2DM. As liver dysfunction is commonly associated with diabetic complications^43^, these findings suggest that aerobic training and vitamin D supplementation could potentially contribute to the management of diabetic liver complications. Also, we showed that the D + AT + HD group had the lowest levels of insulin, glucose, and HOMA-IR (a marker of insulin resistance) among the diabetic groups. This indicates that the intervention may improve glucose metabolism and insulin sensitivity, which are crucial factors in managing diabetic complications such as cardiovascular disease and nephropathy. By enhancing metabolic regulation, aerobic training and vitamin D supplementation could potentially mitigate the risk of these complications in individuals with T2DM. Moreover, the D + AT + HD group exhibited a more profound upregulation of LXRα and PGC-1α proteins in the liver compared to other groups. LXRα and PGC-1α are involved in lipid and glucose metabolism, and their dysregulation is associated with diabetic complications. The upregulation of these markers suggests that the intervention may have a positive impact on molecular pathways related to metabolic regulation, potentially influencing the development and progression of diabetic complications.

Finally, our results indicate a significant association between serum Vit D levels with insulin resistance and visceral fat.There were also significant associations between QUICKI with LXRα and PGC-1α levels. Nonetheless, the increments in PGC-1α and LXRα are significant since they likely explain the reduced glycemic index, liver enzymes and bodyweight in rats with T2DM (Supplementary information).

### Strengths and limitations

The strengths of the present study include the use of an animal model which allows for greater control over environmental factors and the ability to investigate underlying biological mechanisms. Additionally, this study examined the effects of a combination of aerobic exercise and Vit D supplementation, while direct translation may be complex, our study provides valuable insights into the underlying mechanisms of DM. It is important to acknowledge certain limitations that were encountered during the research process. It was not feasible to incorporate the suggested comment from the respected reviewers regarding the use of an established genetic model of type 2 diabetes as a validation study for the observed results, the inclusion of more biochemical parameters, and functional data. Due to various constraints, such as time limitations and availability of resources, the implementation of such a model was not possible within the scope of this study.The possibility that the results may not be directly applicable to humans due to physiological differences between rats and humans, as well as potential ethical concerns related to the use of animal models, are among the limitations of the study. Furthermore, due to the limitations in our study design and scope, we were unable to investigate the upstream and downstream signaling pathways extensively.

### Supplementary Information

Supplementary Information 1.

Supplementary Information 2.

Supplementary Information 3.

Supplementary Information 4.

Supplementary Information 5.

## Data Availability

The datasets generated and analyzed during the current study are not publicly available due to ongoing data analysis but are available from the corresponding author upon reasonable request.

## Abbreviations

Vit D: Vitamin D
AT: Aerobic training
LXRα: Liver X receptors
T2DM: Type 2 diabetes mellitus
PGC1α: Peroxisome proliferator-activated receptor-γ coactivator 1 − α
