# Supplementary figures and images for: Aerobic training with moderate or high doses of vitamin D improve liver enzymes, LXRα and PGC-1α levels in rats with T2DM
Source: Sci Rep. 2024 Mar 17;14:6409. doi: 10.1038/s41598-024-57023-z (PMC10944841; doi:10.1038/s41598-024-57023-z)

**A****LXR $\alpha$** 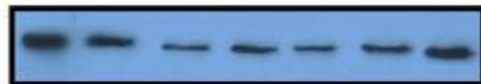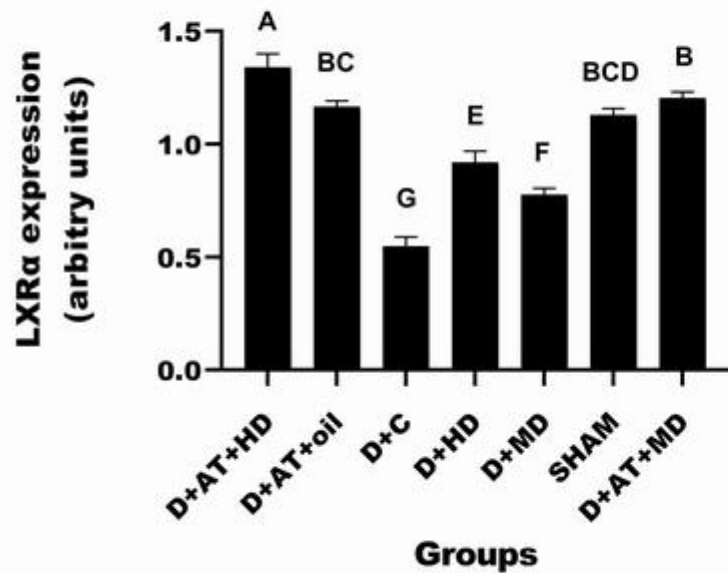**B****PGC-1 $\alpha$** 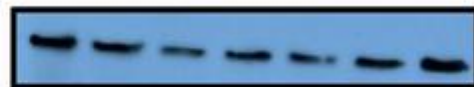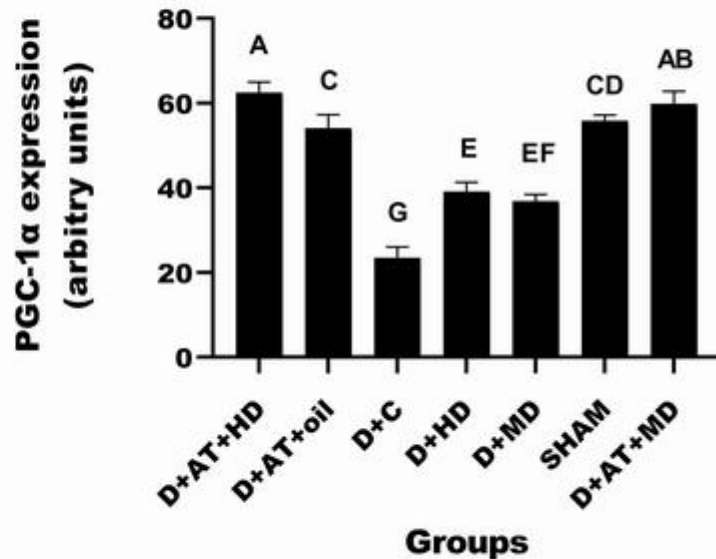

Supplement: Supplementary file 1 — Supplementary Information 1. [file 41598_2024_57023_MOESM1_ESM.pdf]

**SARA**

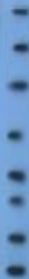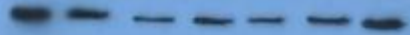

**SARA**

Supplement: Supplementary file 2 — Supplementary Information 2. [file 41598_2024_57023_MOESM2_ESM.pdf]

**A****LXR $\alpha$** 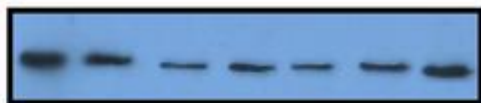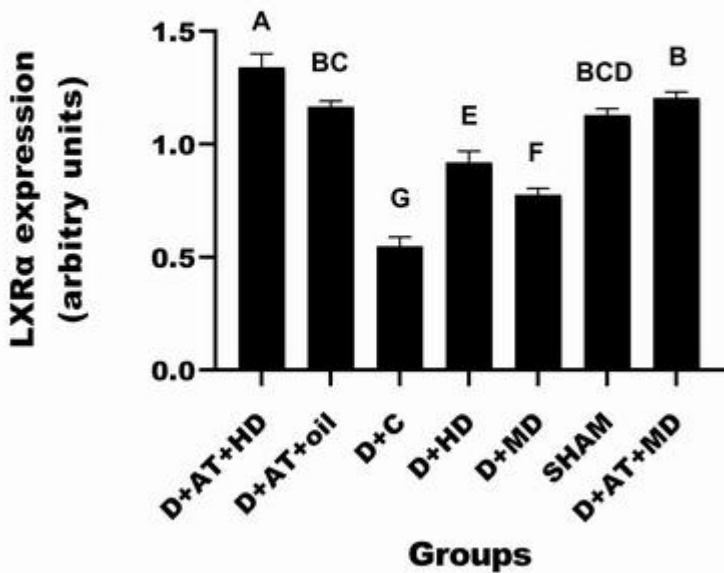

Supplement: Supplementary file 3 — Supplementary Information 3. [file 41598_2024_57023_MOESM3_ESM.pdf]

**B****PGC-1 $\alpha$** 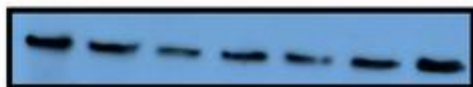**PGC-1 $\alpha$  expression  
(arbitrary units)**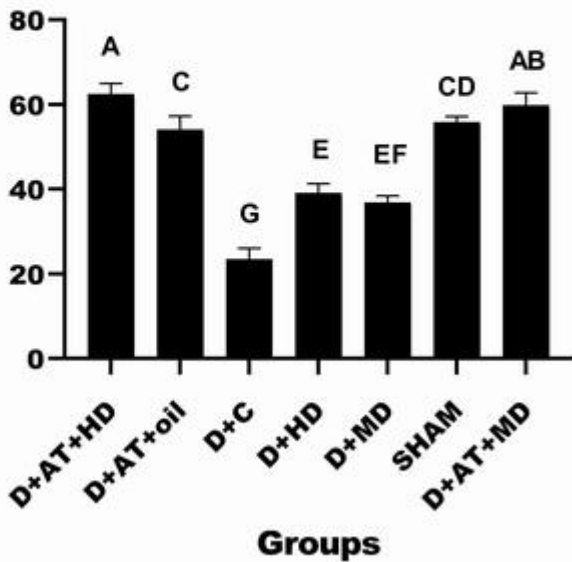

Supplement: Supplementary file 4 — Supplementary Information 4. [file 41598_2024_57023_MOESM4_ESM.pdf]

SARA

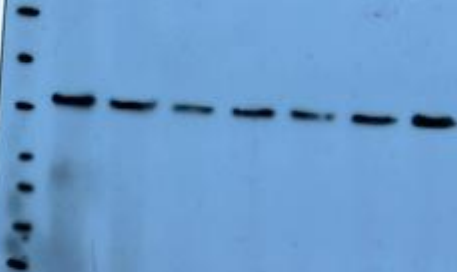

Supplement: Supplementary file 5 — Supplementary Information 5. [file 41598_2024_57023_MOESM5_ESM.pdf]
